# Ecological Momentary Interventions in the Management of Eating Disorders and Obesity: A Systematic Review

**DOI:** 10.1002/eat.70140

**Published:** 2026-06-02

**Authors:** Wenlu Mao, Ricarda Schmidt, Emily K. Presseller, Adrienne Juarascio, Anja Hilbert

**Affiliations:** ^1^ Behavioral Medicine Research Unit, Department of Psychosomatic Medicine and Psychotherapy University Medical Center Leipzig Leipzig Germany; ^2^ Department of Child and Adolescent Psychiatry, Psychotherapy and Psychosomatics University Hospital Leipzig Leipzig Germany; ^3^ Department of Biomedical Data Science Geisel School of Medicine at Dartmouth Hanover New Hampshire USA; ^4^ Department of Psychiatry Geisel School of Medicine at Dartmouth Hanover New Hampshire USA; ^5^ Oregon Research Institute Eugene Oregon USA

**Keywords:** eating disorders, ecological momentary interventions, obesity, systematic review

## Abstract

**Objective:**

This systematic review evaluated the feasibility, efficacy, adherence, and methodological quality of ecological momentary intervention (EMI)–based treatments for individuals with diagnosed eating disorders (EDs) and/or obesity.

**Method:**

Eight electronic databases were systematically searched from inception to January 2026. Randomized, non‐randomized, and uncontrolled studies evaluating EMI‐based treatments for individuals with diagnosed EDs and/or obesity were included. Data on intervention characteristics, clinical and behavioral outcomes, feasibility, acceptability, and adherence were extracted. Risk of bias was assessed using RoB 2 and ROBINS‐I, and findings were synthesized narratively.

**Results:**

Fourteen studies met inclusion criteria (eight obesity‐focused, six ED‐focused), including nine randomized controlled trials. In ED studies, EMI‐augmented interventions were associated with significant within‐group reductions in binge‐eating frequency and ED psychopathology. However, superiority over active cognitive‐behavioral therapy‐based controls was inconsistent and primarily observed during maintenance phases. Obesity‐focused trials demonstrated small to moderate within‐group improvements in weight‐related outcomes, with generally small and non‐sustained between‐group effects. Feasibility and acceptability were generally high, although retention and engagement varied across studies. Risk of bias was low in most randomized trials but higher in non‐randomized studies.

**Discussion:**

EMIs appear to be a feasible and acceptable approach for extending evidence‐based treatments for EDs and obesity into daily life. Current evidence suggests that EMIs may exert a beneficial adjunctive effect by enhancing engagement and improving short‐term behavioral outcomes. However, their impact on long‐term clinical outcomes remains limited and inconsistent, underscoring the necessity for conducting rigorously designed clinical trials with extended follow‐up periods.

## Summary

- Digital tools, such as ecological momentary interventions (EMIs), are increasingly being employed in daily life to support the treatment of eating disorders and obesity.
- This systematic review found that while EMI can enhance patient engagement and improve short‐term behavioral outcomes, evidence for sustained clinical benefits remains insufficient.
- Further trials with more rigorous designs and extended follow‐up periods are required to clarify the long‐term efficacy of EMI and its clinical utility.

## 1 Introduction

Eating disorders (EDs), including anorexia nervosa (AN), bulimia nervosa (BN), and binge‐eating disorder (BED), are serious mental disorders characterized by abnormal eating and/or weight control behaviors (American Psychiatric Association 2013; Treasure et al. 2020). EDs affect approximately 8.4% of women and 2.2% of men, with lifetime prevalence estimated at 4% to 9% (Galmiche et al. 2019). EDs are associated with high mortality and disability rates, and cognitive behavioral therapy (CBT) remains one of the most well‐established psychological treatments, benefiting approximately 40%–67% of patients (Cuijpers et al. 2025; Hilbert et al. 2017). Because BN and BED involve recurrent overeating in terms of binge eating and are often accompanied by overweight and obesity, EDs and obesity may involve partially overlapping behavioral and self‐regulatory processes relevant to eating and weight regulation (Heaner and Walsh 2013; Masheb et al. 2011). Obesity, defined as excessive or abnormal accumulation of body fat (body mass index, BMI ≥ 30 kg/m^2^), has reached pandemic proportions, affecting about 16% of the adults globally in 2022 (NCD Risk Factor Collaboration [NCD‐RisC] 2024) and over 40% of adults in the United States (NCD‐RisC 2024; Stierman et al. 2021). Obesity is strongly linked to numerous negative physical and psychological consequences (Ward et al. 2022), and treatment primarily relies on behavioral and lifestyle interventions, with pharmacological or bariatric surgery reserved for more severe cases (Perdomo et al. 2023). While EDs and obesity are distinct conditions, they may involve partially overlapping behavioral and self‐regulatory processes relevant to intervention design, including responsiveness to affective states, cravings, and sensitivity to environmental cues (Fairburn 2008; Smith and Juarascio 2019; Stinson et al. 2022). Importantly, obesity is increasingly conceptualized as a complex chronic disease characterized by excessive adiposity and influenced by biological, psychological, and environmental factors, and should not be considered equivalent to an ED (Hruby and Hu 2015; Rubino et al. 2025). Examining these conditions within a shared intervention framework may help identify common and distinct targets for scalable, real‐time interventions across related but separate clinical populations (Nahum‐Shani et al. 2017).

Mobile technology, especially smartphones, increasingly provides accessible platforms for assessments and interventions in EDs and obesity, enabling self‐monitoring, psychoeducation, and behavior change (Couto and de Almeida 2025; Hsu et al. 2022; Z. McClure et al. 2024). Ecological momentary assessment (EMA) refers to the repeated, real‐time assessment of symptoms, behaviors, and contextual factors in individuals' natural environments and has demonstrated feasibility for capturing daily behavioral patterns while reducing recall bias (Schembre et al. 2018; Stinson et al. 2022). However, EMA is fundamentally an assessment method rather than an intervention (Smith and Juarascio 2019). In contrast, ecological momentary interventions (EMIs) involve the delivery of therapeutic support or intervention components in daily life, ideally in a timely and context‐responsive manner (Nahum‐Shani et al. 2015, 2017). Unlike approaches that rely solely on self‐monitoring or non‐responsive scheduled messaging, EMIs are designed to provide in‐the‐moment support embedded within individuals' everyday contexts, with the goal of intervening at critical moments when maladaptive behaviors are most likely to occur (Nahum‐Shani et al. 2017; Smith and Juarascio 2019). An advanced form, just‐in‐time adaptive interventions (JITAIs), use real‐time data and algorithms to provide personalized interventions at the most relevant moments, adapting dynamically to individuals' changing internal and external contexts (Doherty et al. 2020; Nahum‐Shani and Murphy 2025). Gamification—the use of game‐like elements (e.g., rewards or challenges)—has also been applied in some EMI‐based interventions to enhance engagement and motivation (Sardi et al. 2017).

Recent work has systematically examined the use of EMA in obesity, highlighting the value of real‐time data in capturing dynamic behavioral and contextual processes (e.g., Rapelli et al. 2025). While such research provides important insights into momentary determinants of eating behaviors, it primarily focuses on assessment rather than intervention (Shiffman et al. 2008). EMIs aim to translate these insights into real‐time therapeutic support delivered in individuals' natural environments (Heron and Smyth 2010; Nahum‐Shani et al. 2017). In this sense, EMA can inform the identification of high‐risk states (e.g., negative affect, cravings, environmental triggers), whereas EMIs represent an attempt to intervene at these critical moments through timely, context‐sensitive strategies (Nahum‐Shani et al. 2017; Smith and Juarascio 2019). More recently, Koh et al. (2025) conducted a scoping review of JITAIs for weight management among adults with overweight and obesity. Given the different and broad operationalization of JITAIs adopted in that review, some overlap in obesity‐focused studies was expected, with eight obesity‐related studies overlapping across the two reviews despite substantial differences in scope and inclusion criteria. Whereas Koh et al. primarily focused on describing JITAI characteristics and delivery approaches, the present review applied more explicit operational criteria for EMI classification and specifically synthesized treatment‐focused EMI interventions across ED and obesity populations, with additional emphasis on feasibility, adherence, clinical outcomes, and methodological quality. Overall, existing reviews in this area have primarily focused on EMA, broader digital health interventions, or obesity and EDs separately, with limited synthesis of treatment‐focused EMI interventions across these distinct clinical populations.

The present systematic review evaluated the feasibility, efficacy, adherence, and the methodological quality of EMI‐based interventions in EDs (including AN, BN, and BED) and obesity in order to provide a comprehensive understanding of EMIs and their potential in the treatment of EDs and obesity.

## 2 Method

### 2.1 Search Strategy

A comprehensive literature search was conducted across eight electronic databases: PubMed, PsycINFO, MEDLINE, Scopus, Web of Science, Google Scholar, EMBASE, and the Cochrane Central Register of Controlled Trials (CENTRAL). The search strategy employed focused on EMIs and digital health interventions in EDs and obesity, using the following search terms: ((“ecological momentary intervention” OR “EMI” OR “eHealth” OR “mobile health” OR “mHealth” OR “digital health interventions” OR “smartphone‐supported intervention” OR “just‐in‐time adaptive intervention” OR “adaptive intervention” OR “real‐time monitoring” OR “real time data capture” OR “self‐monitoring intervention” OR “telehealth” OR “mobile phone intervention” OR “text message intervention”) AND (“eating disorder” OR “disordered eating” OR “anorexia nervosa” OR “bulimia nervosa” OR “binge‐eating disorder” OR “AN” OR “BN” OR “BED” OR “binge eating” OR “obesity” OR “overweight” OR “weight loss” OR “weight management”)). These terms were searched within titles, abstracts, and keywords across databases.

The search covered all literatures from database inception through January 2026. No language restrictions were applied during the search. All retrieved references were imported into Zotero reference management software (Corporation for Digital Scholarship, Vienna, VA, United States). After automatic removal of duplicates, the remaining records were screened for eligibility based on their titles and abstracts. Subsequently, full texts of eligible studies were assessed according to the predefined inclusion and exclusion criteria by two independent raters with graduate‐level training. Discrepancies were resolved through discussion and, when necessary, through consultation within the research team until consensus was reached.

The entire study selection process was conducted in accordance with the PRISMA 2020 guidelines and was illustrated in a PRISMA flow diagram (Figure 1; Page et al. 2021). The review protocol was preregistered in the PROSPERO database (registration number: CRD42024605769).

**FIGURE 1:**
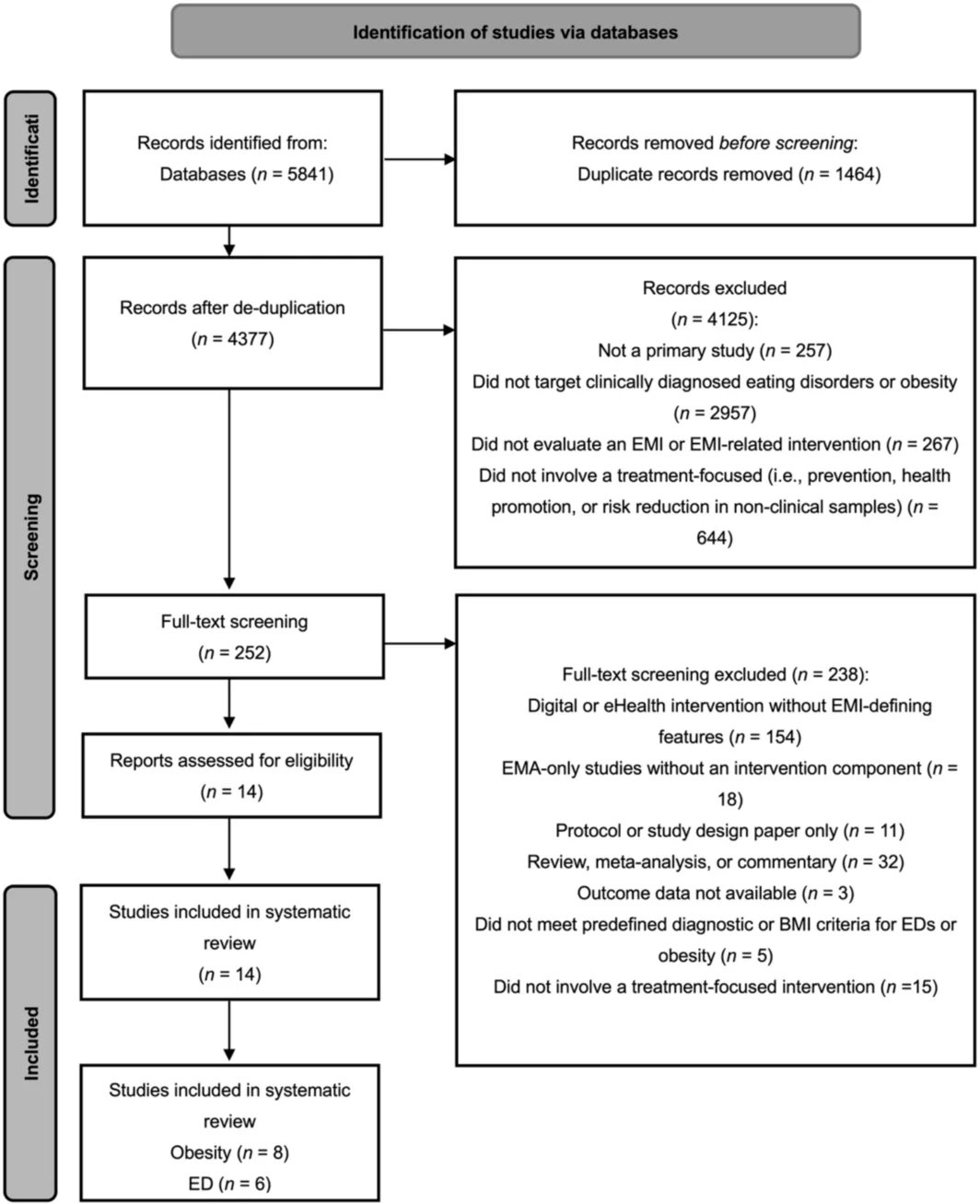
PRISMA flow diagram of study selection. *Note:* Adapted from Page et al. (2021).

### 2.2 Study Selection

Studies were included if they met the following criteria: (1) ED diagnosis as defined by the Diagnostic and Statistical Manual of Mental Disorders, fifth edition (DSM‐5) (American Psychiatric Association 2013), including AN, BN, and BED full‐threshold or of low frequency and/or limited duration, established through clinical interview or diagnostic questionnaire; or obesity (and higher‐risk overweight), defined as a BMI ≥ 27 kg/m^2^ based on objective measurement or self‐report, consistent with clinical guideline recommendations indicating that structured treatment or lifestyle modification should be considered from this threshold onward, particularly in the presence of health risks or comorbidities (Deutsche Adipositas‐Gesellschaft [DAG] e.V. 2024); (2) EMIs delivered during the active treatment phase; (3) interventions met operational criteria for EMIs, including the delivery of therapeutic feedback, behavioral support, or intervention content beyond assessment alone via mobile or digital platforms in real‐world, momentary contexts; (4) interventions incorporated real‐time prompts, behavioral tracking with feedback; interactive mobile or digital applications, or context‐sensitive intervention delivery; (5) momentary data collection with repeated, real‐time assessments (e.g., daily or weekly) to capture emotional, behavioral, or clinical patterns related to EDs or obesity. (6) randomized controlled trials (RCTs), non‐randomized controlled trials (non‐RCTs), or uncontrolled intervention designs.

Interventions focused solely on passive self‐monitoring or non‐responsive scheduled messaging, without the delivery of therapeutic feedback, behavioral support, or intervention content, were excluded.

### 2.3 Data Extraction

Data from all included studies were extracted using a standardized sheet, covering study and participant characteristics, ED and obesity assessments, EMI methodology, and outcomes. A full version of the data extraction sheet is provided in the online supplement (Table S1). Operational definitions of key methodological and feasibility‐related terms (e.g., feasibility, acceptability, adherence and retention), as well as commonly used abbreviations, are provided in the online supplement (Table S2).

### 2.4 Risk of Bias Assessment

For risk of bias assessment, the included RCTs were evaluated using the Cochrane Risk of Bias tool (RoB 2) for RCTs, which evaluates five domains of bias: the randomization process, deviations from intended interventions, missing outcome data, measurement of the outcome, and selection of the reported result (Sterne et al. 2019). For non‐RCTs of interventions, the Risk Of Bias In Non‐randomized Studies—of Interventions (ROBINS‐I) tool was used, assessing biases across seven domains, including confounding, selection of participants, classification of interventions, deviations from intended interventions, missing data, measurement of outcomes, and selection of the reported result (Sterne et al. 2016). All risk‐of‐bias assessments were conducted independently by two reviewers, with discrepancies resolved through discussion until consensus was reached.

### 2.5 Strategy for Data Synthesis

Given the heterogeneity of EMIs in terms of intervention features, populations, intervention components, and outcome measures, a narrative synthesis was conducted to examine their use in EDs and obesity treatment. Extracted data were organized according to five prespecified domains: study characteristics, participant characteristics, ED and obesity assessment, EMI methodology and clinical outcomes. Detailed descriptions of the extracted variables were provided in the online Supporting Information (Table S1). Descriptive statistics were used to summarize demographic characteristics, clinical features, key intervention components, and indicators of engagement or adherence across included studies. Clinical outcomes underwent qualitative synthesis based on reported standardized effect sizes, statistical significance, and authors' interpretations. When effect sizes were not reported, we calculated standardized effect sizes (Cohen's *d*) based on means, standard deviations, and sample sizes provided in the original articles. Where feasible, recurring patterns and sources of heterogeneity were identified by comparing findings across diagnostic groups (EDs vs. obesity) and study designs (randomized vs. non‐randomized trials).

### 2.6 Additional Transparency Statements

#### 2.6.1 Use of Generative AI Tools

Generative artificial intelligence (ChatGPT, OpenAI, GPT‐5.2; https://chat.openai.com/) was used solely for language editing. All study design, data extraction, analysis, and interpretation were conducted by the authors.

### 2.7 Lived Experience Statement

Persons with lived experience of EDs or obesity were not involved in the design, conduct, or reporting of this systematic review.

## 3 Results

### 3.1 Study Selection

A total of 5841 records were identified through the systematic search. After the removal of 1464 duplicate records, 4377 unique records remained for title and abstract screening. At the end of title and abstract screening, 4125 articles were removed, leaving 252 articles eligible for full‐text screening. In total, 14 studies met all inclusion criteria and were included in the present systematic review. Eight studies focused on obesity, while six studies focused on EDs. Among the 14 studies, nine were RCTs, three were uncontrolled intervention studies, one used a single‐arm withdrawal design, and one employed a single‐case alternating‐treatment design. All eligible studies were published in English. A detailed overview of the study selection process is provided in Figure 1.

### 3.2 Characteristics of Participants

Across included studies, participant characteristics differed between ED‐ and obesity‐focused trials, with ED samples generally smaller and younger, and obesity samples larger and more heterogeneous in age and BMI. Across both conditions, samples were predominantly White and female, and ethnicity reporting was limited. The 14 included studies comprised a total of 959 participants receiving EMI‐based intervention, with sample sizes ranging from 5 to 199. Participant characteristics were summarized for studies targeting EDs and obesity (see Table 1).

**TABLE 1:** Participant characteristics of included studies targeting eating disorders and obesity.

Study (Author, year)	Study design	Country	Sample size, *n (Intervention/Control*)	Age (years), mean (SD)	Female, *n* (%)	Race, *n* (%)	Ethnicity	Mean BMI (kg/m^2^)	Education (SES indicator)	Condition	Baseline assessments of ED and obesity
Eating disorders											
Hilbert et al. (2025)	RCT	Germany	25 (13/12)	42.5 (12.5)	9 (69.0)	Not reported	Not reported	39.1 (SD = 9.0)	≥ 12 years, *n* = 4 (31.0%)	BED	EDE (DSM‐5)
Juarascio et al. (2021)	Non‐RCT	USA	5 (5/0)	35.6 (6.8)	4 (80.0)	White (80.0%, *n* = 4), Multiracial (20.0%, *n* = 1)	Not reported	31.2 (SD = 6.8)	Not reported	BN	EDE (DSM‐5)
Juarascio et al. (2022)	Non‐RCT	USA	30 (30/0)	37.1 (12.2)	26 (86.7)	White (90.0%, *n* = 27)	Hispanic/Latino (7%, *n* = 2), Non‐Hispanic/Latino (93%, *n* = 28)	29.5 (SD = 5.2)	Not reported	BED, BN	EDE (DSM‐5)
Juarascio, Presseller, Srivastava, et al. (2023)	RCT	USA	56 (29/27)	38.1 (13.6)	25 (86.2)	White (62.1%, *n* = 18), African American (17.2%, *n* = 5), Asian (10.3%, *n* = 3), Multiracial or other (20.6%, *n* = 6)^a^	Hispanic/Latino (3%, *n* = 1), Non‐Hispanic/Latino (90%, *n* = 26), Not reported (7%, *n* = 2)	29.9 (SD = 6.9)	Not reported	BN; BN‐spectrum OSFED	EDE (DSM‐5)
Shapiro et al. (2010)	Non‐RCT	USA	31 (31/0)	26.3 (8.6)	31 (100.0)	White, (93.6%, *n* = 29), African American (3.2%, *n* = 1), Asian (3.2%, *n* = 1)	Not reported	Not reported	Not reported	BN	SCID (DSM‐IV), EDI, Binge‐Purge Questionnaire, NEQ
Shingleton et al. (2016)	Non‐RCT	USA	12 (12/0)	21.5 (2.4)	10 (83.0)	White (66.7%, *n* = 8)	Not reported	20.0 (SD = 2.9)	Not reported	AN, subclinical AN, BN	EDE, SCID‐I, RMQ, EDE‐Q
Obesity											
Burke et al. (2022)	RCT	USA	502 (251/251)	45.0 (14.3)	200 (79.7)	White (84.1%, *n* = 211)	Not reported	33.7 (SD = 3.9)	Education year, *M* = 16.5 (SD = 2.9)	Overweight or obesity (BMI ≥ 27 kg/m^2^)	Objectively measured (height, weight)
Carter et al. (2013)	RCT	UK	43^b^	41.2 (8.5)	33 (76.7)	White (100%, *n* = 43)	Not reported	33.7 (SD = 4.2)	College degree, *n* = 31 (72.1%)	Overweight or obesity (BMI ≥ 27 kg/m^2^)	Objectively measured (height, weight)
Donaldson et al. (2014)	RCT	UK	34 (17/17)	58.3 (12.1)	10 (58.8)	Not reported	Not reported	35.6 (SD = 5.0)	Not reported	Overweight or obesity (BMI ≥ 30 kg/m^2^, or ≥ 28 kg/m^2^ with comorbidities)	Objectively measured (height, weight)
Forman et al. (2019)	RCT	USA	181 (119/62)	47.2 (13.3)	100 (84.0)	White (73.5%, *n* = 133)	Not reported	34.5 (SD = 5.7)	Not reported	Overweight or obesity (BMI ≥ 25.0 kg/m^2^)^c^	Objectively measured (height, weight)
Mundi et al. (2015)	Non‐RCT	USA	30 (30/0)	41.3 (11.4)	27 (90.0)	Not reported	Not reported	46.3 (SD = 7.4)	College degree, *n* = 14 (46.7%)	Medically complicated obesity (pre‐bariatric surgery)	Objectively measured (height, weight)
Spring et al. (2024)	RCT	USA	400 (199/201)	40.5 (11.2)	305 (76.4)	White (66.5%, *n* = 266), Black (20.3%, *n* = 81), Asian (4.3%, *n* = 17), Other (1.3%, *n* = 5)	Hispanic/Latino (13%, *n* = 25)	34.4 (SD = 4.3)	College degree, *n* = 349 (87.3%)	Obesity (BMI ≥ 30 kg/m^2^)	Objectively measured (height, weight)
Svetkey et al. (2015)	RCT	USA	122^b^	29.2 (4.2)	84 (68.9)	White (55.7%, *n* = 68), Black (34.4%, *n* = 42), Other (9.8%, *n* = 12)	Hispanic/Latino (7%, *n* = 9)	35.7 (SD = 8.2)	College degree or higher, *n* = 83 (68.0%)	Overweight or obesity (BMI ≥ 25.0 kg/m^2^)^c^	Objectively measured (height, weight)
van Beurden et al. (2019)	RCT	UK	88 (58/30)	46.7 (13.6)	37 (64.0)	White (92.9%, *n* = 52), Other (7.1%, *n* = 4)	Not reported	32.8 (SD = 5.6)	College degree, *n* = 39 (70.0%)	Overweight or obesity (BMI ≥ 25.0 kg/m^2^)^c^	Objectively measured (height, weight)

*Note:* All BMI values reported in kg/m^2^.

Abbreviations: AN = anorexia nervosa; BED = binge eating disorder; BMI = body mass index; BN = bulimia nervosa; EDE (DSM‐V) = eating disorder examination (Diagnostic and Statistical Manual of Mental Disorders, fifth edition); EDE‐Q = Eating Disorder Examination‐Questionnaire; EDI = Eating Disorders Inventory; *M* = mean; *n* = number; NEQ = Night Eating Questionnaire; Non‐RCT = non‐randomized controlled study; OSFED = other specific feeding and eating disorder (OSFED included BN of low frequency and/or limited duration); RCT = randomized controlled study; RMQ = Readiness and Motivation Questionnaire; SCID = Structured Clinical Interview for DSM‐IV, eating disorders modules; SD = standard deviation; SES = socioeconomic status; WC = waist circumstance.

^a^ Percentages may exceed 100% because participants could endorse more than one racial category.

^b^ Sample size refers to the group that received the ecological momentary intervention (EMI), and does not necessarily reflect the total number of participants enrolled in the study. For Carter et al. (2013), the smartphone group is reported here (*n* = 43); diary (*n* = 43) and website (*n* = 42) groups served as comparison conditions. For Svetkey et al. (2015), the cell phone group (CP; *n* = 122) is reported; the control group (*n* = 123) and the personal coaching plus smartphone self‐monitoring group (PC; *n* = 120) were comparison conditions and are not shown.

^c^ Although some studies used a BMI threshold of ≥ 25 kg/m^2^, the mean BMI of participants was consistently in the obese range, and interventions explicitly targeted obesity treatment.

Among the studies targeting EDs (*n* = 6), three primarily focused on BN (Juarascio et al. 2021; Juarascio, Presseller, Srivastava, et al. 2023; Shapiro et al. 2010), including one study that also enrolled participants with BN‐spectrum other specified feeding or eating disorders (OSFED). One study targeted BED (Hilbert et al. 2025), one included both AN and BN (Shingleton et al. 2016), and one enrolled a mixed BN/BED sample (Juarascio et al. 2022). Sample sizes within EMI intervention conditions were relatively small (range: 5–31, *M* = 20.0, SD = 11.3), and participants were predominantly women (range: 69%–100%, *M* = 84.2%, SD = 10.1%). Mean ages ranged from 21.5 to 42.5 years (*M* = 33.5, SD = 7.9). Participants' BMI (kg/m^2^) varied widely across studies, from 20.0 to 39.1 kg/m^2^ (*M* = 30.1 kg/m^2^, SD = 7.8), with the highest values observed in a trial on BED where many participants had comorbid obesity (Hilbert et al. 2025).

In studies targeting obesity (*n* = 8), sample sizes were generally larger than in ED trials, ranging from 17 to 199 (*M* = 104.9, SD = 84.6). Participants' mean age was typically in the middle to older adult range (range: 29.2–58.3 years, *M* = 43.7, SD = 8.2). Across studies, female participants represented the majority of the samples (ranging from 59% to 90%, *M* = 74.8%, SD = 10.4%). Among studies reporting race (*n* = 6), the proportion of White participants ranged from 55.7% to 100%. All participants met criteria for overweight or obesity, typically defined as BMI ≥ 30 kg/m^2^, though two studies used a cutoff of ≥ 27 kg/m^2^ with or without comorbidities (Carter et al. 2013; Donaldson et al. 2014), spanning 33.7 to 46.3 kg/m^2^ (*M* = 35.8 kg/m^2^, SD = 4.3).

### 3.3 Characteristics of Interventions

The EMIs employed across the included studies demonstrated considerable variability in terms of format, content, frequency, and methods of delivery (Table 2).

**TABLE 2:** Characteristics of EMI interventions.

Study (Author, Year)	EMI modality	Delivery mode	Content components	Frequency	Duration of intervention	Duration of follow‐up	Control intervention(s)
Eating disorders							
Hilbert et al. (2025)	Smartphone app (trEATsmart)	App with JITAIs, reminders, push notifications and self‐monitoring; web‐based clinician portal; adjunct to CBT	Electronic self‐monitoring of eating behavior, mood, body image, physical activity, and weight Algorithm‐based JITAIs triggered by momentary data (e.g., weighing, inactivity) targeting normalization of eating behavior, emotion and impulse regulation, and body image Therapist‐defined and automated reminders; clinician portal for tailoring and monitoring	Daily self‐monitoring; event‐based JITAIs triggered by momentary data; real‐time delivery	4 months	3 months	Manualized CBT (16 sessions/4 months); no app, JITAIs, or automated feedback
Juarascio et al. (2021)	Smartphone app (CBT+)	App with automated JITAIs, reminders, push notifications, and self‐monitoring; web‐based clinician portal; adjunct to CBT	Electronic self‐monitoring of eating behaviors and mood; algorithm‐based JITAIs for target engagement triggered by entries or inactivity Data‐driven personalized CBT skill exercises delivered in‐app	Daily real‐time self‐monitoring during meals, urges, or mood changes; event‐based JITAIs triggered by user entries; immediate delivery	16 weeks	Not reported	Not reported
Juarascio et al. (2022)	Smartphone app (SenseSupport)	App with automated JITAIs, reminders, push notifications, and real‐time self‐monitoring; CGM integration and clinician portal	Electronic self‐monitoring of eating behavior, mood, and ED behaviors CGM‐based detection of prolonged fasting and algorithm‐triggered JITAIs promoting regular eating Therapist review of CGM and app data during sessions	Real‐time, event‐based JITAIs triggered by CGM‐detected fasting and self‐monitoring entries	12 weeks	Not reported	Not reported
Juarascio, Presseller, Srivastava, et al. (2023)	Smartphone app (CBT+)	CBT+ app with digital self‐monitoring, in‐app JITAIs, and clinician‐defined notifications; adjunct to CBT	Electronic self‐monitoring (eating behavior, ED symptoms, skill use) In‐app JITAIs triggered by entries or inactivity Push notifications for self‐monitor and clinician‐defined notifications CBT skill interventions with rationale and instructions	Daily self‐monitoring; event‐ and inactivity‐based JITAIs (≥ 5 h); immediate delivery	16 weeks	3 months	Manualized CBT‐E (16 sessions) + CBT+ app for self‐monitoring only; no JITAIs or push notifications
Shapiro et al. (2010)	Smartphone–based ecological momentary self‐monitoring and intervention	Automated SMS feedback; adjunct with group CBT	Nightly self‐monitoring (binge, purge, urges) Feedback triggered by daily self‐monitoring entries or missing data Personalized CBT‐based feedback messages (reinforcement and motivation)	Nightly self‐monitoring; feedback triggered by daily entries or missing data; real‐time delivery	12 weeks	12 weeks	Not reported
Shingleton et al. (2016)	Motivational text messages via mobile phone	Personalized motivational SMS from pretreatment MI session; adjunct to CBT	Personalized motivational messages based on MI session Messages addressing ED‐related cognitions and behaviors Tailoring based on pretreatment motivational interviewing session	Daily motivational SMS	8 weeks	Not reported	Not reported
Obesity							
Burke et al. (2022)	SMART smartphone app; Fitbit food diary; Fitbit activity tracker	Therapist‐based feedback messages and notifications via app, self‐monitoring via Fitbit	Self‐monitoring of dietary intake, weight, PA Feedback triggered by self‐monitoring data or inactivity Tailored feedback messages addressing one behavior at a time Therapist‐guided tailoring	Daily self‐monitoring; event‐ and inactivity‐based feedback; real‐time delivery	12 months	Not reported	Self‐monitoring only, no tailored feedback
Carter et al. (2013)	My Meal Mate (MMM) smartphone app	Smartphone‐based intervention	Self‐monitoring of diet and physical activity Scheduled feedback delivery Goal‐oriented feedback messages supporting weight‐loss adherence Tailoring based on self‐monitoring data	Self‐monitoring with weekly summaries; time‐based feedback; scheduled delivery	6 months	Not reported	Website access or paper diary self‐monitoring
Donaldson et al. (2014)	SMS‐based EMI	SMS reminders and feedback with bidirectional patient–practitioner messaging	Self‐monitoring of lifestyle behaviors (diet, PA) Feedback triggered by twice‐weekly submissions or non‐response Personalized goal‐based feedback and encouraging messages Bidirectional patient–practitioner messaging	Twice‐weekly self‐monitoring; event‐based feedback triggered by submissions or non‐response; scheduled delivery	12 weeks	Not reported	No text messages, reminders, or feedback
Forman et al. (2019)	Smartphone app (OnTrack)	Smartphone app with JITAIs, push notifications, real‐time risk alerts, and self‐monitoring; adjunct to digital Weight Watchers program	Real‐time lapse risk prediction using machine learning Momentary, trigger‐matched behavioral micro‐interventions Self‐monitoring of lapses and triggers Personalized adaptive algorithm (group + individual data)	Daily semi‐random trigger monitoring (6 prompts/day); event‐based JITAIs triggered by algorithm‐detected lapse risk; immediate real‐time delivery	10 weeks	Not reported	Weight Watchers digital weight loss program alone
Mundi et al. (2015)	Smartphone app with EMA/EMI messaging	Algorithm‐generated tailored feedback messages; adjunct with video‐based education modules	Self‐monitoring prompted via EMA of eating and PA; EMI feedback triggered by EMA responses Tailored supportive and congratulatory messages Algorithm‐based branching logic	EMA prompts delivered multiple times daily; event‐based EMI feedback triggered by EMA responses; immediate delivery	12 weeks	Not reported	Not reported
Spring et al. (2024)	Smartphone‐based intervention with wireless feedback system	Fitbit tracker and scale synced to app; optional notifications; 10–15 min coaching calls via dashboard data	Self‐monitoring of diet, PA, and weight Continuous feedback based on device‐linked data Automated feedback and supportive alerts Optional coach‐supported tailoring	Daily self‐monitoring via devices; continuous data‐driven feedback; continuous delivery	12 weeks	Not reported	WFS only; no notifications or coaching
Svetkey et al. (2015)	Smartphone‐based intervention	App for intervention delivery and self‐monitoring; protocol‐driven self‐management prompts	Self‐monitoring of weight, diet, and physical activity Protocol‐driven prompts delivered multiple times daily Goal‐setting challenges and automated support Rule‐based personalization	Continuous self‐monitoring; protocol‐driven prompts multiple times daily; scheduled delivery	12 months	Not reporetd	Three handouts on healthy eating and physical activity from Eat Smart Move More North Carolina; no digital intervention, or self‐monitoring
van Beurden et al. (2019)	Smartphone‐based app (ImpulsePal)	Smartphone‐based self‐delivered EMI; personalized inhibition training by food category	Self‐monitoring of food‐related impulses Interventions triggered by momentary need or context Inhibition training and visuospatial loading for impulse control Personalized training by food category	Daily self‐monitoring; event‐based EMI delivery; real‐time delivery	3 months	3 months	Waiting list control; no app access during study

Abbreviations: CBT= cognitive behavioral therapy; CBT‐E = enhanced cognitive behavioral therapy; CGM = continuous glucose monitoring; ED = eating disorder; EMA = ecological momentary assessment; EMI = ecological momentary intervention; JITAIs = just‐in‐time adaptive interventions; MI = motivational interviewing; PA = physical activity; SMS = short message service; WFS = wireless feedback system.

#### 3.3.1 EDs

Across ED studies, EMIs were consistently delivered as adjuncts to CBT and typically involved smartphone‐based platforms providing real‐time feedback and skills‐based support. Four studies used smartphone apps incorporating self‐monitoring and JITAI‐based support (Hilbert et al. 2025; Juarascio et al. 2021, 2022; Juarascio, Presseller, Srivastava, et al. 2023), whereas two used short message service (SMS) or text‐based mobile platforms (Shapiro et al. 2010; Shingleton et al. 2016). All ED‐focused EMIs involved daily use, with intervention duration ranged from 8 to 16 weeks (*M* = 13.3, SD = 3.3). Self‐monitoring was a universal and typically covered ED symptoms, mood, and/or contextual triggers. JITAI‐based interventions reinforced CBT‐related skills such as regular eating, coping with urges, cognitive restructuring, and emotion regulation, while the continuous glucose monitoring (CGM)‐triggered intervention targeted dietary restriction through physiology‐informed feedback.

#### 3.3.2 Obesity

In contrast to ED studies, obesity‐focused EMIs were more heterogeneous in format and delivery, and were typically implemented as standalone behavioral weight‐loss (BWL) programs rather than adjuncts to psychotherapy. Regarding intervention formats, five studies utilized smartphone apps (Burke et al. 2022; Carter et al. 2013; Forman et al. 2019; Mundi et al. 2015; van Beurden et al. 2019), two studies applied smartphone‐based platforms primarily relying on SMS or app‐mediated prompts (Donaldson et al. 2014; Svetkey et al. 2015), and one study implemented a smartphone‐based wireless feedback system (WFS), defined as an integrated system combining a smartphone app with connected devices (e.g., wearable activity trackers and digital scales) to deliver automated feedback (Spring et al. 2024). Intervention frequency varied, ranging from daily self‐monitoring with multiple feedback messages per day (e.g., Burke et al. 2022; Forman et al. 2019; Mundi et al. 2015; Spring et al. 2024; Svetkey et al. 2015; van Beurden et al. 2019) to weekly prompts (e.g., Carter et al. 2013; Donaldson et al. 2014). The duration of the intervention ranged from 10 to 52 weeks (*M* = 22.3, SD = 16.5). All obesity‐focused EMIs were grounded in BWL principles, integrating dietary intake, physical activity (PA), and weight self‐monitoring with goal setting and feedback. Feedback was typically tailored based on participants' prior intervention usage patterns, self‐monitoring entries, inactivity, or deviations from dietary and PA goals. Across obesity studies, interventions commonly combined dietary and PA self‐monitoring with tailored feedback, reminders, goal‐setting, and, in some cases, machine learning‐driven or location‐sensitive support. Compared to ED‐focused EMIs, which were consistently delivered as adjuncts to CBT, obesity‐focused interventions were more often implemented as standalone behavioral programs with greater variability in delivery format and intervention duration.

### 3.4 Quantitative Summary of EMI Components

Across studies, EMI components showed both shared and condition‐specific patterns, with self‐monitoring and tailored feedback widely implemented across both ED and obesity interventions, while more advanced adaptive features (e.g., JITAIs) were more common in ED‐focused studies. A quantitative summary of EMIs features is presented in Table 3.

**TABLE 3:** Quantitative summary of EMI components.

Study	Self‐monitoring	Automated feedback	Tailored messages	Reminders/prompts	JITAIs	Push notifications	Gamification	Location‐specific goal primes	Clinician‐guided
Eating disorders									
Hilbert et al. (2025)	✓	✓	✓	✓	✓	✓			✓
Juarascio et al. (2021)	✓	✓	✓	✓	✓	✓			✓
Juarascio et al. (2022)	✓	✓	✓	✓	✓	✓			✓
Juarascio, Presseller, Srivastava, et al. (2023)	✓	✓	✓	✓	✓	✓			✓
Shapiro et al. (2010)	✓	✓	✓	✓					✓
Shingleton et al. (2016)	✓	✓	✓						✓
Obesity									
Burke et al. (2022)	✓	✓	✓	✓		✓			✓
Carter et al. (2013)	✓	✓	✓	✓					
Donaldson et al. (2014)	✓	✓	✓	✓					✓
Forman et al. (2019)	✓	✓	✓	✓	✓	✓			
Mundi et al. (2015)	✓	✓	✓	✓			✓		
Spring et al. (2024)	✓		✓	✓		✓			✓
Svetkey et al. (2015)	✓	✓	✓	✓		✓			
van Beurden et al. (2019)	✓	✓	✓	✓			✓	✓	

*Note:* Components were coded based on explicit reporting in the intervention description. “Self‐monitoring” referred to digital recording of dietary intake, weight, physical activity, mood, body image or eating disorder symptoms, as applicable. “Automated feedback” denoted system‐generated feedback messages without manual input from clinicians. “Tailored messages” referred to individualized content adjusted to patient data or goals. “Reminders/prompts” included SMS, app notifications, or scheduled cues to record data or practice skills. “Push notifications” indicated smartphone alerts triggered either automatically (e.g., inactivity) or pre‐scheduled by a clinician. “Gamification” referred to reward or scoring features integrated into the intervention. “Location‐specific goal primes” indicated goal priming via GPS‐based location services (e.g., geofencing, geo‐caching). “Clinician‐guided” referred to interventions involving direct professional involvement.

Abbreviations: EMI = ecological momentary intervention; JITAIs = just‐in‐time adaptive interventions.

For EDs, self‐monitoring, automated feedback, and tailored messages were included in all six studies (6/6, 100%). Reminders or prompts—primarily cues to complete self‐monitoring entries or practice specific therapeutic skills—were reported in 5/6 (83%), except for Shingleton et al. (2016). More advanced features, including JITAIs, defined by algorithm‐driven decision rules that dynamically tailor intervention content based on momentary states or behaviors, and push notifications, were implemented in 4/6 (67%) studies (Hilbert et al. 2025; Juarascio et al. 2021, 2022; Juarascio, Presseller, Srivastava, et al. 2023). No studies utilized gamification or location‐specific goal primes. However, all studies were embedded within clinician‐guided treatment programs, with EMI components delivered automatically as adjuncts to ongoing care.

For obesity, self‐monitoring, tailored messages, and push notifications were included in all eight studies (8/8, 100%). All studies except Spring et al. (2024) included automated feedback (7/8, 88%). One study employed the JITAI framework (1/8, 13%; Forman et al. 2019). Push notifications were implemented in three trials (4/8, 50%; Burke et al. 2022; Forman et al. 2019; Spring et al. 2024; Svetkey et al. 2015), and guidance by a clinician was also provided in three trials (3/8, 38%; Burke et al. 2022; Donaldson et al. 2014; Spring et al. 2024). Gamification was supported in 2/8 (25%) studies. Only one study (1/8, 13%) employed location‐specific goal primes, which identified high‐risk environments and delivered prompts based on participants' real‐time locations (van Beurden et al. 2019).

Most ED and obesity EMI interventions were grounded in established behavior change frameworks, including CBT (Hilbert et al. 2025; Juarascio et al. 2021, 2022; Juarascio, Presseller, Srivastava, et al. 2023; Shapiro et al. 2010), motivational interviewing (Shingleton et al. 2016; Svetkey et al. 2015), and self‐regulation or dual‐process models (Burke et al. 2022; Carter et al. 2013; Mundi et al. 2015; Spring et al. 2024; van Beurden et al. 2019). Across both ED and obesity studies, a subset of interventions further implemented JITAI architectures to deliver context‐sensitive behavioral support (Forman et al. 2019; Hilbert et al. 2025; Juarascio et al. 2021, 2022; Juarascio, Presseller, Srivastava, et al. 2023). All interventions targeting EDs were delivered as augmentation to traditional individual (Hilbert et al. 2025; Juarascio et al. 2021; Juarascio et al. 2022; Juarascio, Presseller, Srivastava, et al. 2023; Shingleton et al. 2016) or group‐based (Shapiro et al. 2010) behavioral therapy sessions. In contrast, obesity‐focused EMI interventions were primarily delivered as standalone BWL programs, with digital tools serving as the central mode of treatment rather than adjuncts to face‐to‐face care.

### 3.5 Intervention Outcomes

Across both ED and obesity studies, EMI‐based interventions were generally associated with improvements over time, although the magnitude and consistency of between‐group effects varied across conditions and outcome types. For ED trials, outcomes focused on binge‐eating frequency and/or abstinence, dietary restriction, and global ED psychopathology, while obesity trials focused on weight‐related outcomes (e.g., body weight, BMI, waist circumstance), dietary quality, PA, and metabolic risk factors (e.g., glucose level, lipid profiles). Assessment timepoints ranged from post‐intervention to longer‐term follow‐ups (6–12 months). A detailed outcome summary is presented in Table 4.

**TABLE 4:** Outcome measures and treatment effects.

Study	Study design	Outcome measures	Assessment time points	Feasibility/Adherence	Acceptability/Engagement	Clinical efficacy	Retention
Eating disorders							
Hilbert et al. (2025)	RCT, CBT+ trEATsmart app (“SmartCBT) versus CBT	Primary: Feasibility (CBT sessions attendance; app use metrics), OBEs at post‐treatment (EDE) Secondary: OBEs at 3‐month follow‐up, ED psychopathology (EDE‐Q), anxiety (GAD‐7), self‐efficacy (GSES), emotion regulation (DERS), clinical impairment (CIA), weight‐related quality of life (IWQOL), anthropometric outcomes (BMI, WHR)	Baseline, mid‐treatment (2 months), post‐treatment (4 months), 3‐month follow‐up	High usability and perceived practicality of the trEATsmart app (8.3 ± 1.1/10) Particularly useful for promoting regular eating, identifying triggers, and increasing physical activity	App used on 66% ± 29% of therapy days (~3 entries/day); Mean of 37 ± 24 JITAIs delivered, with 92% implemented	Across both treatment arms, all psychometric outcomes showed large improvements from baseline to 3‐month follow‐up, whereas BMI increased overall Greater reduction in objective binge episodes with SmartCBT compared to CBT at post‐treatment (*d* = −0.9) This effect was no longer significant after controlling for gender and was not observed at 3‐month follow‐up Small between‐group differences favored SmartCBT for BMI and waist‐to‐hip ratio at post‐treatment and follow‐up (*β* = −0.1 to −0.2), whereas CBT showed slightly greater improvements in self‐efficacy at both time points (*β* = 0.2–0.3) Remission rates were lower in SmartCBT compared to CBT (46% vs. 75% at 4 months)	9/14 (64%) retained at post‐treatment
Juarascio et al. (2021)	Pilot study, non‐RCT	Primary: Feasibility and acceptability (phone interviews and weekly questionnaires) Secondary: ED psychopathology and QOL (EDE, QOLI)	Baseline, mid‐treatment (Week 8), post‐treatment (week 16)	High app use (86.1% ± 7.5% of days) Mean 3.1 ± 0.7 entries/day Mean 2.1 ± 0.5 notifications and 0.8 ± 0.5 interventions/day 91.9% of interventions targeted dietary restraint	For the CBT+ app, median usefulness rating 5/6; participants received an average of 2.1 ± 0.5 notifications and 0.8 ± 0.5 interventions per day	Decreases in OBEs and global ED symptoms at post‐treatment Large within‐group effects (*d* > 0.8)	3/5 (60%) retained at mid‐treatment
Juarascio et al. (2022)	Non‐RCT, single‐arm withdrawal (ABAB) design	Primary: Feasibility, acceptability, dietary restriction (fasting ≥ 5 h; EDE‐Q restraint items), and ED behaviors Secondary: Binge episodes, compensatory behaviors, and EDE global and subscale scores	Baseline, mid‐treatment (weeks 4 and 8), post‐treatment (Week 12)	Mean CGM data capture = 67.4% (range 16.8–92.2); 33% achieved 80% capture Frequent app‐ and sensor‐related issues were reported (participants: 93% and 37%; therapists: 88% and 25%, respectively)	High acceptability of CGM and system usability (≥ 79%–100% favorable; ≥ 92% ease/usefulness); 79% likely to use or recommend the system; Qualitative feedback emphasized supportive accountability, helpful reminders to eat, and learning physiological responses to food	Significant pre‐ to post‐treatment reductions in binge episodes, compensatory behaviors, and EDE global and subscale scores with medium‐to‐large within‐group effects (*d* = 0.68–2.07) Faster reductions in weekly compensatory behaviors during JITAI‐off versus JITAI‐on periods No significant difference in weekly binge‐eating change between JITAI‐on and JITAI‐off periods Small improvements in dietary‐restriction indicators during JITAI‐on periods (target engagement effects)	25/30 (83%) retained at post‐treatment
Juarascio, Presseller, Srivastava, et al. (2023)	RCT, CBT+ app with JITAIs‐on vs. CBT+ app with JITAIs‐off	Primary: Feasibility and acceptability (7‐point Likert scale questionnaire and interviews) Secondary: Changes in ED symptoms (EDE)	Baseline, mid‐treatment, post‐treatment (Week 16), 3‐month follow‐up	Good adherence to self‐monitoring (*M* = 3.0, SD = 1.5 entries/day), aligning with the intended three daily entries Adherence declined slightly after mid‐treatment	High acceptability in both conditions; JITAIs‐On condition rated easier to use (medium effect size)	EMI‐augmented CBT (JITAIs‐On) produced greater reductions in binge eating and ED symptoms at post‐treatment (Week 16) compared to control Large effect sizes observed at post‐treatment At follow‐up, binge‐eating reduction was maintained in JITAIs‐On, whereas 76% of JITAIs‐Off participants relapsed	26/29 participants (90%) completed post‐treatment assessment
Shapiro et al. (2010)	Uncontrolled intervention study	Primary: Feasibility and adherence to self‐monitoring Secondary: Acceptability (0–10 Likert scale questionnaire) and BN symptoms change (EDI, Binge‐Purge Questionnaire, NEQ)	Baseline, Post‐treatment (Week 12), Follow‐up (Week 24)	High adherence to self‐monitoring (87%)	Positive ratings across domains (expectations: *M* = 7.1 ± 2.0/10; recommendation: *M* = 7.9 ± 1.6/10; willingness to participate again: *M* = 7.7 ± 2.8/10; enjoyment of text messaging: *M* = 6.3 ± 2.4/10), except for self‐monitoring forms which were rated as average (*M* = 5.0 ± 2.4/10)	Significant reductions in binge eating, purging, ED psychopathology, depressive symptoms, and night eating from baseline to post‐treatment Improvements maintained at 24‐week follow‐up	15/31 (48%) completed treatment and post‐treatment assessment
Shingleton et al. (2016)	Uncontrolled intervention study, single‐case alternating treatment	Primary: Feasibility and acceptability (five Likert‐scale questionnaire); dietary restraint, restriction and motivation (EDE, SCID‐I, RMQ, EDE‐Q, daily food records)	Baseline, post‐treatment (12 weeks)	High compliance (91.5% daily entries, 87.4% food logs)	Overall positive acceptability (*M* = 7.1 ± 2.4/10); Self‐monitoring rated lowest (*M* = 5.3 ± 2.3/10); Enjoyment of text messaging rated moderate (*M* = 6.3 ± 3.1/10)	Reductions in dietary restraint and improvements in motivation (decreased precontemplation, increased action/confidence) No significant effects on kilocalorie intake or dietary restraint when examined as direct text‐message effects	12/13 (92%) completers
Burke et al. (2022)	RCT, self‐monitoring (SM) versus self‐monitoring plus feedback (SM + FB)	Primary: Weight loss at 6, 12 months (weight); Secondary: Weight loss status (≥ 5% versus < 5% of baseline weight)	Baseline, 6 months, 12 months	Median 42.2% of feedback messages opened; SM + FB group showed higher adherence to calorie goals than SM alone (*β* = 4.43)	Not reported	Both SM and SM + FB groups achieved significant weight loss from baseline No significant between‐group difference in percentage or absolute weight loss at 12 months Between‐group effect negligible (*d* = 0.06)	202/251 (81%) retained at 12 months
Carter et al. (2013)	RCT, smartphone versus website versus paper diary	Primary: Feasibility and acceptability (questionnaire); Secondary: Anthropometric (weight, BMI, BF)	Baseline, 6 weeks, 6 months	Highest self‐monitoring adherence in the smartphone group (mean 92 ± 67 days) Lower adherence in paper diary (29 ± 39 days) and website groups (35 ± 44 days)	Smartphone group reported greater satisfaction than diary and website groups; At 6 weeks, 91.2% of smartphone users reported satisfaction or high satisfaction	Greater weight loss in the smartphone group (−4.6 kg) compared to paper diary (−1.3 kg) Website group showed intermediate weight loss (−2.9 kg) ≥ 5% weight loss achieved by 37% (smartphone), 28% (diary), and 17% (website); between‐group differences not significant Standardized effects were small (e.g., smartphone vs. website: *d* ≈0.15)	Smartphone: 40/43 (93%) at 6 months; Website: 19/42 (45%); Paper diary: 20/43 (47%)
Donaldson et al. (2014)	RCT, LEAP Beep intervention vs. control	Primary: Effectiveness and acceptability (survey) Secondary: Changes in health variables (weight, WC, IWQOL), lifestyle factors (steps, fruit/vegetable intake, breakfast consumption)	Baseline, 12 weeks	High satisfaction reported by all intervention participants Improved motivation commonly endorsed	Not reported	Significant reductions in weight (−2.3 kg), BMI (−1.5 kg/m^2^), and waist circumference (−3.7 cm) versus control No significant between‐group differences in quality of life, anxiety, or depressive symptoms Between‐group BMI effect small (*d* = 0.16)	14/17 (82%) of intervention group completed satisfaction survey
Forman et al. (2019)	RCT, Weight Watcher + OnTrack app versus WW	Primary: Percent weight loss Secondary: Satisfaction, lapse frequency, intervention engagement, algorithm accuracy (sensitivity/specificity)	Baseline, 10 weeks	62.9% (SD = 28.11) of prompted lapse‐trigger entries completed Mean 118.5 (SD = 52.8) risk alerts delivered 46.9% (SD = 26.2) of alerts opened; declined over time Mean 7.4% (SD = 10.1) of full interventions accessed Algorithm sensitivity 69.2% (SD = 24.9) and specificity 83.8% (SD = 10.4)	Overall satisfaction high and did not differ by treatment condition; OnTrack interventions rated helpful/accurate 72.8% of the time; Greater satisfaction for Beyond the Scale versus Freestyle within OnTrack condition (*d* ≈0.62)^a^	No main effect of treatment condition on percent weight loss Significant diet type × condition interaction (*η* ^2^ ≈0.05, small to moderate interaction effect) Beyond the Scale: higher weight loss with WW + OnTrack (4.7%) than WW alone (2.6%)^a^ Freestyle: lower weight loss with WW + OnTrack (2.9%) than WW alone (4.5%)^a^ No overall superiority of OnTrack for weight loss	OnTrack + WW: 104/119 (87%) complete treatment; WW: 56/62 (90%) completed treatment
Mundi et al. (2015)	Uncontrolled intervention study	Satisfaction and perceived usefulness (electronic survey), weight change, PA (IPAQ‐SF), EMA response (rate and timing)	During pre‐surgery preparation period (≈207 days mean)	High satisfaction (80%) and perceived usefulness Majority reported healthier choices and greater connection to care	31% of EMA prompts answered within 60 min; Higher EMA responsiveness associated with greater exercise confidence	Mean presurgical weight loss of 7.3 kg (5.6% of baseline) BMI decreased significantly (small effect, *d* = 0.15) Vigorous physical activity increased (*d* = 0.50)	20/30 (67%) completed
Spring et al. (2024)	RCT, wireless feedback system (WFS) versus WFS+ coaching	Primary: Weight change difference at 6 months Secondary: Weight change at 3 and 12 months Exploratory: ≥ 5% weight loss, adherence (questionnaire).	Baseline, 3 months, 6 months, 12 months	High adherence to self‐monitoring and coaching in the WFS + coaching group Behavioral adherence (dietary and weight self‐monitoring) significantly higher than WFS‐only	Not reported	Greater weight loss in WFS + coaching versus WFS at 6 months (−4.8 vs. −2.8 kg; difference −2.0 kg) Higher proportion achieving ≥ 5% weight loss at 3 and 6 months; no group difference at 12 months Between‐group effect size negligible (*d* = 0.05), indicating minimal clinical impact despite statistical significance	371/400 (93%) at 3 months, 342/400 (86%) at 6 months, 326/400 (82%) at 12 months
Svetkey et al. (2015)	RCT, cell phone (CP) versus personal coaching (PC) versus control	Primary: Weight change at 24 months Secondary: Weight change at 6, 12 months; change in PA Exploratory: ≥ 5% weight loss (24 months); adherence	Baseline, 6 months, 12 months, 24 months	Intervention adherence declined over time in both CP and PC conditions CP self‐weighing decreased from 4.0 times/week (months 1–6) to 2.1 times/week (months 13–24)	Not reported	PC achieved greater weight loss than control at 6 months (−1.92 kg) and than both control and CP at 12 months (−2.19 kg) No significant between‐group differences at 24‐month follow‐up CP did not differ from control at any time point Effect sizes were small (*d* = 0.08), consistent with modest mean differences	306/356 (86%) retained at 24 months
van Beurden et al. (2019)	RCT, ImpulsePal app versus control	Primary: Feasibility, usability and satisfaction (5‐point Likert response scales questionnaire), weight change at 1, 3 months; Secondary: Unhealth snack food/drink consumption (7‐day FFQ), Overeating episodes and LOC (EDE‐Q)	Baseline, 1 month, 3 months	High satisfaction (93%) and ease of use (98%) Mean usability ratings high (4.7 ± 0.5/5)	Not reported	Greater weight loss in intervention vs. control at 1 month (*d* = 0.2); effect attenuated at 3 months Greater reductions in loss of control and uncontrolled eating days in intervention group (small effects, *d* ≈0.2) Improvements in eating behavior largely maintained at follow‐up	74/88 (84%) at 1 month; 67/88 (76%) at 3 months

Abbreviations: BED = binge eating disorder; BF = body fat; BMI = body mass index; BN = bulimia nervosa; CGM = continuous glucose monitoring; CIA = clinical impairment assessment; CID‐I = Structured Clinical Interview for Axis I DSM‐IV disorders; DERS = difficulties in emotion regulation scale; ED = eating disorder; EDE = Eating Disorder Examination; EDE‐Q = Eating Disorder Examination Questionnaire; EDI = Eating Disorders Inventory‐II; EMA = ecological momentary assessment; FFQ = Food Frequency Questionnaire; GAD‐7 = Generalized Anxiety Disorder 7; GSES = General Self‐efficacy Scale; IPAQ‐SF = International Physical Activity Questionnaire Short Form; IWQOL = Impact of Weight on Quality of Life; JITAI = just‐in‐time adaptive intervention; LOC = loss of control; mo = months; NEQ = Night Eating Questionnaire; OBE = objective binge episode; PA = physical activity; QOLI = Quality of Life Inventory; RCT = randomized controlled trial; RMQ = Readiness and Motivation Questionnaire; SMS = short message service; WC = waist circumstance; WHR = waist‐to‐hip ratio; WW = weight watcher.

^a^ Beyond the scale and freestyle refer to different versions of the weight watchers dietary program introduced during the trial; assignment was not randomized.

#### 3.5.1 Intervention Outcomes in Eating Disorders

Across randomized and non‐randomized studies, EMI‐augmented interventions were generally associated with improvements in binge‐eating behaviors and ED psychopathology over time, with strong within‐group effects but limited and inconsistent evidence for superiority over active control conditions.

Among RCT with active controls, Juarascio, Presseller, Srivastava, et al. (2023) found that both the JITAI‐On condition and the self‐monitoring only control (JITAI‐Off) demonstrated reductions in binge‐eating frequency and ED psychopathology at post‐treatment, indicating large within‐group effects. Between‐group differences were not observed at post‐treatment; however, a notable pattern emerged during the follow‐up phase, with symptom reductions sustained at follow‐up only in the JITAI‐On condition, whereas the JITAI‐Off group demonstrated a 76% increase in binge‐eating frequency from post‐treatment to follow‐up. Assessment completion at post‐treatment was high in both groups (26/29, 89.7%; 23/26, 88.5%).

In a pilot RCT, Hilbert et al. (2025) reported that both SmartCBT (individual CBT augmented with EMI components) and CBT‐alone groups showed reductions over time in binge‐eating frequency and improvements in ED psychopathology, reflecting large within‐group treatment effects. At post‐treatment, greater reductions in binge‐eating frequency were observed in the SmartCBT group compared with CBT alone, with a large between‐group effect (*d* = −0.9). However, these differences were attenuated after adjustment for baseline imbalances and were not maintained at follow‐up. Importantly, improvements over time were observed in both arms for primary and secondary outcomes.

Non‐randomized studies (Juarascio et al. 2021, 2022; Shapiro et al. 2010; Shingleton et al. 2016) similarly reported within‐group improvements in binge‐eating behaviors and ED psychopathology, alongside high feasibility and acceptability of EMI components (Table 4).

#### 3.5.2 Intervention Outcomes in Obesity

Across obesity trials, interventions were associated with modest improvements in weight and related behavioral outcomes over time, with generally small and often non‐sustained between‐group differences.

In large RCT, Spring et al. (2024) and Svetkey et al. (2015) reported statistically significant short‐term differences favoring EMI‐enhanced or coaching‐supported conditions; however, between‐group differences were small in magnitude and were not sustained at longer‐term follow‐up. Similarly, a JITAI‐based RCT (Forman et al. 2019) found no overall between‐group weight‐loss advantage of EMI, but showed greater effectiveness across diet prescriptions (*η* ^*2*^ ≈0.05), with greater weight loss under EMI in one diet condition (Beyond the Scale) and the opposite pattern in the other (Freestyle).

In addition, Carter et al. (2013), Donaldson et al. (2014), Burke et al. (2022), and van Beurden et al. (2019) primarily evaluated component comparisons or passive‐control designs (e.g., smartphone‐based versus paper‐ or web‐based self‐monitoring, or self‐monitoring alone versus self‐monitoring with tailored feedback). Across these trials, between‐group differences in weight‐related outcomes were consistently small, with reported standardized effect sizes typically falling in the very small‐to‐small range (approximately *d* = 0.06–0.20), despite within‐group improvements over time in all study arms.

The only uncontrolled obesity trial, Mundi et al. (2015), provided preliminary feasibility evidence, with within‐group improvements in presurgical weight loss and PA; however, the absence of a control condition limits causal inference, and findings should be interpreted as exploratory (Table 4).

Overall, while ED studies tended to show stronger improvement in proximal behavioral outcomes (e.g., binge‐eating frequency), obesity studies more consistently demonstrated modest changes in distal anthropometric outcomes, with limited sustained between‐group differences. Across both ED and obesity trials, assessment completion and engagement with EMI components were generally moderate to high; however, attrition and app usage patterns varied substantially across studies. Detailed indicators of feasibility, adherence, and usage are summarized in Table 4 and are further considered in the Discussion.

### 3.6 Risk of Bias

Overall, methodological quality was higher in randomized than non‐randomized studies. Among the nine RCTs, the overall risk of bias was generally low (Figure 2), with both ED‐focused RCTs and two obesity‐focused RCTs rated as low risk across domains (Burke et al. 2022; Hilbert et al. 2025; Juarascio, Presseller, Srivastava, et al. 2023; Spring et al. 2024).

**FIGURE 2:**
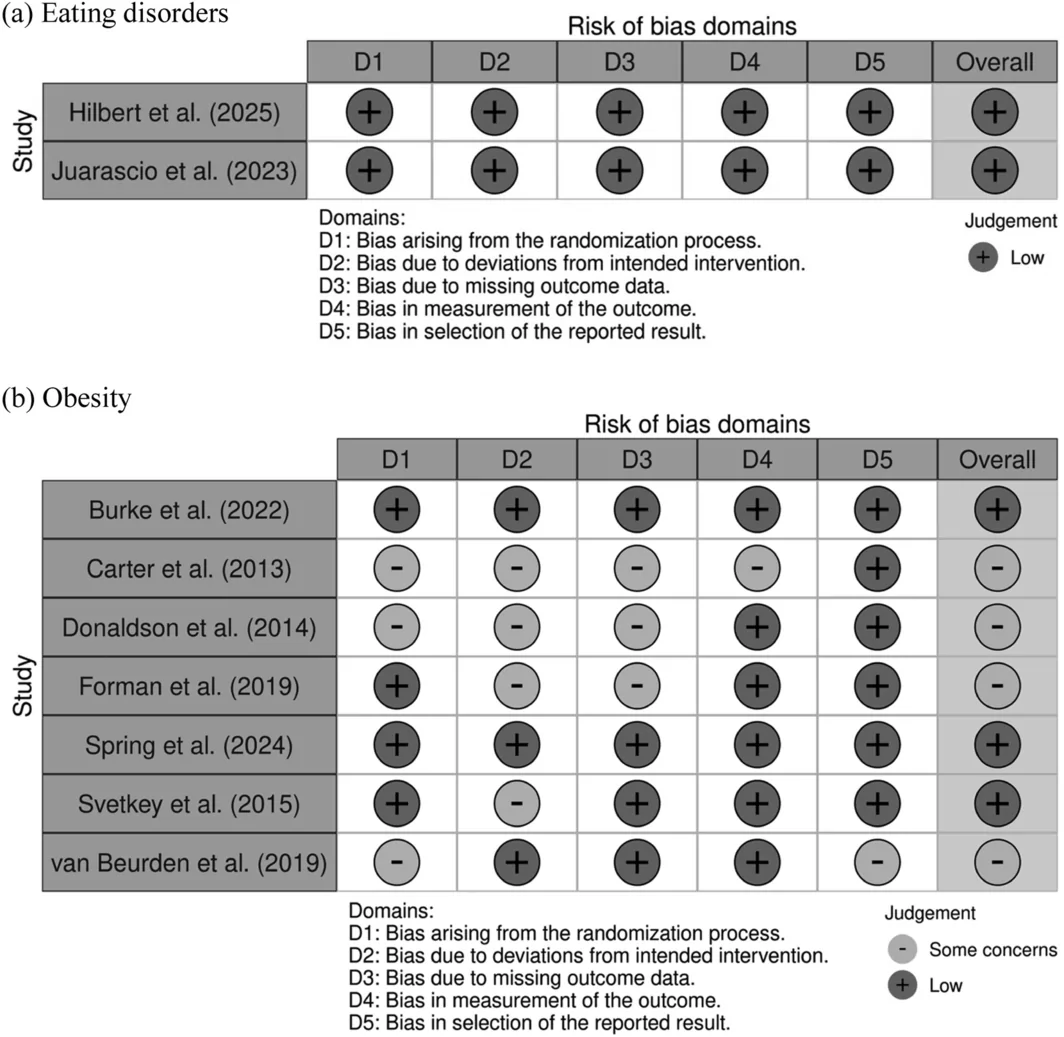
Risk of bias assessment for randomized controlled trials in ED (RoB 2). (a) Eating disorders. (b) Obesity. *Note:* Risk of bias was evaluated using the RoB 2 tool across five domains. Symbols denote risk judgments: + = low risk; − = moderate risk; × = serious risk.

Across the five included non‐randomized studies, overall risk of bias was variable but generally higher (Figure 3). For ED studies, Juarascio et al. (2021, 2022) were rated as having serious overall risk, primarily due to confounding and missing outcome data, whereas Shapiro et al. (2010) and Shingleton et al. (2016) demonstrated moderate overall risk, largely reflecting unblinded and self‐reported outcome assessment. The obesity study by Mundi et al. (2015) was also judged to have serious risk due to confounding and incomplete follow‐up. Across studies, bias in outcomes measurement was commonly rated as moderate, consistent with limited blinding.

**FIGURE 3:**
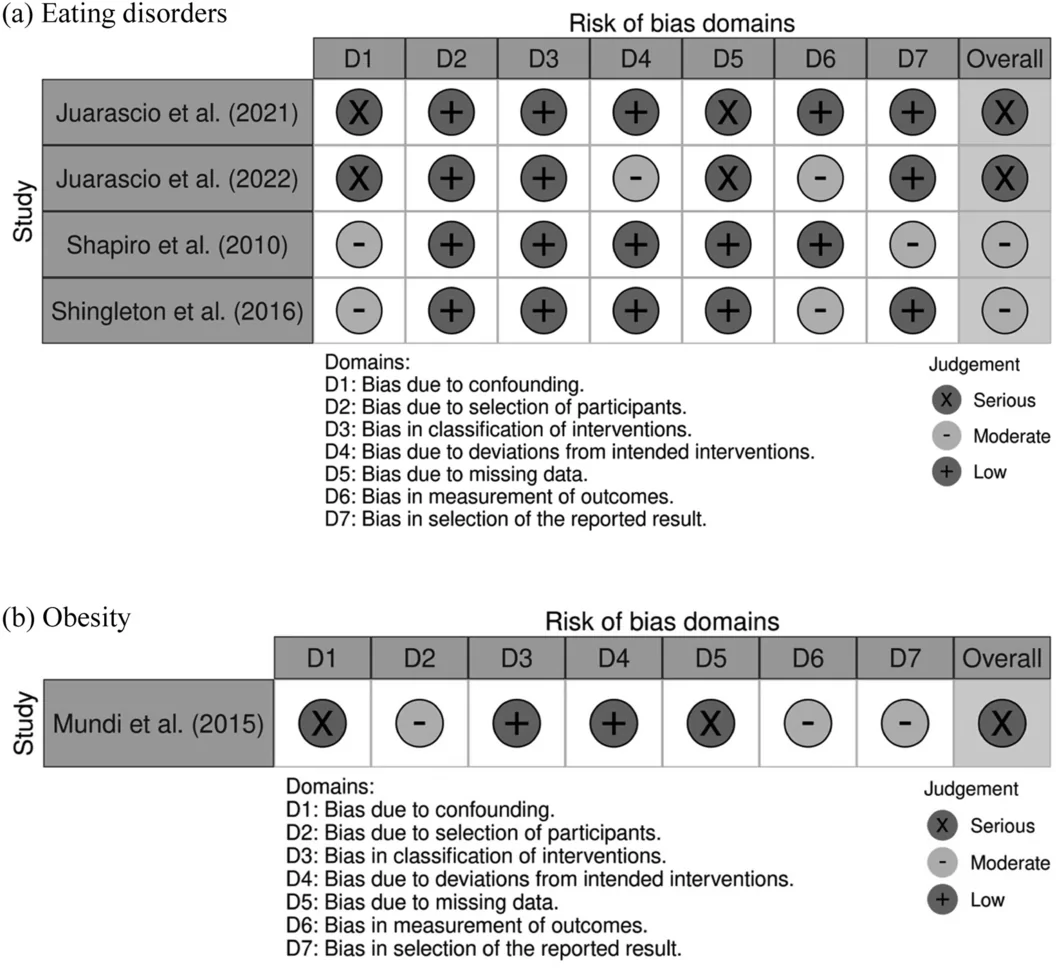
Risk of bias assessment for non‐randomized studies (ROBINS‐I). (a) Eating disorders. (b) Obesity. *Note:* Risk of bias for non‐randomized studies was evaluated using the ROBINS‐I tool across seven domains. Symbols denote risk judgments: + = low risk; − = moderate risk; × = serious risk.

## 4 Discussion

### 4.1 Summary of Results

This systematic review provides a comprehensive synthesis of EMIs targeting EDs (AN, BN, BED) and obesity, highlighting efforts to bridge traditional therapy with everyday life. Across the 14 included studies, EMIs were delivered predominantly as adjuncts to established treatments—most commonly CBT or structured BWL programs—while true stand‐alone applications remained limited.

Among individuals with EDs receiving EMIs—most often used adjunctively to CBT—reductions were observed in binge‐eating episodes and restrictive eating behaviors, alongside improvements in selected secondary processes such as emotion regulation capabilities and treatment motivation (Hilbert et al. 2025; Juarascio et al. 2021, 2022; Juarascio, Presseller, Srivastava, et al. 2023; Shapiro et al. 2010; Shingleton et al. 2016). However, consistent superiority over control conditions was not observed across outcomes or time points (Hilbert et al. 2025; Juarascio, Presseller, Srivastava, et al. 2023), potentially reflecting strong active comparators and limited statistical power due to small sample sizes. Benefits were more evident for symptom maintenance and treatment engagement—particularly in adaptive or just‐in‐time EMI formats—than for immediate post‐treatment symptom reduction.

For individuals with obesity, EMIs were typically embedded within theory‐driven BWL programs rather than delivered as standalone treatments. Across studies, EMIs produced small‐to‐moderate improvements in weight‐related outcomes during active treatment, with less consistent maintenance at follow‐up. However, between‐group differences were generally small or negligible, and adaptive EMI approaches demonstrated context‐dependent rather than consistently superior effects (Burke et al. 2022; Carter et al. 2013; Forman et al. 2019; Spring et al. 2024; Svetkey et al. 2015; van Beurden et al. 2019). Overall, current evidence suggests that EMIs primarily extend behavioral support into daily life, with limited additional weight‐loss benefit beyond standard behavioral interventions.

### 4.2 Applicability, Feasibility, and Adherence

The applicability of EMIs was reflected in participants' engagement, satisfaction, and continued use across studies. Across ED‐focused trials, EMIs were generally perceived as acceptable and useful for supporting self‐regulation and symptom monitoring in daily life, consistent with broader EMI research in behavioral and menta health contexts demonstrating the acceptability of momentary, context‐sensitive interventions (Nahum‐Shani et al. 2017). Engagement levels were generally moderate to high across EMI‐augmented CBT and JITAI‐based interventions for BN and BEN, consistent with engagement patterns reported in broader mobile health and EMI research across health behavior domains (Hilbert et al. 2025; Juarascio et al. 2021; Juarascio, Presseller, Srivastava, et al. 2023; Juarascio, Presseller, Trainor, et al. 2023; Klasnja et al. 2018). However, sustained engagement remained challenging, with technology‐related burden, repetitive self‐monitoring, technical difficulties, and time demands commonly reported as barriers to continued use and adherence (Carter et al. 2013; Juarascio et al. 2021, 2022; Svetkey et al. 2015). Earlier ED‐focused studies similarly suggested that repetitive self‐monitoring was often perceived as less engaging than feedback or therapeutic support components, highlighting the importance of balancing monitoring intensity with perceived usefulness and user burden in EMI design (Folkersma et al. 2021; Heron and Smyth 2010; Shapiro et al. 2010; Shingleton et al. 2016). Overall feasibility appeared favorable within outpatient ED settings; however, evidence regarding the maintenance of engagement beyond active treatment remained limited. Where follow‐up data were available (e.g., Hilbert et al. 2025; Juarascio, Presseller, Srivastava, et al. 2023; Shapiro et al. 2010), adherence generally declined once ongoing intervention support ended, highlighting challenges in sustaining behavioral engagement over time.

Across obesity‐focused studies, EMI‐based interventions similarly demonstrated favorable applicability and engagement across behavioral weight‐management contexts. Personalized feedback, reminders, and real‐time monitoring appeared to support self‐regulation and participation in daily self‐management routines (Donaldson et al. 2014; Mundi et al. 2015; Spring et al. 2024). Consistent with findings in ED‐focused studies, engagement commonly declined over time, particularly in interventions involving prolonged monitoring periods or high‐frequency prompts. Importantly, greater intervention intensity did not consistently correspond to superior clinical outcomes, suggesting that more intensive monitoring may increase participant burden without necessarily improving effectiveness. This observation aligns with broader EMA/EMI literature indicating that excessive monitoring demands may undermine long‐term adherence and user satisfaction (Pratap et al. 2020; Shiffman et al. 2008; Turner et al. 2019).

Whereas applicability and feasibility appeared broadly similar across ED‐focused and obesity‐focused interventions, retention tended to be higher in obesity‐focused studies, with post‐treatment completion ranging from approximately 67% to 93%. Retention in ED trials ranged from about 48% to 92%, comparable to or somewhat higher than rates reported in traditional ED treatment (15%–65%; Hoste et al. 2007; Schnicker et al. 2013). This retention rate is also comparable to or higher than that observed for other chronic conditions such as breast cancer (approximately 45%, Min et al. 2014), depression (approximately 66%, Arean et al. 2016), or diabetes (approximately 70%; Lyles et al. 2011). This difference may reflect factors such as older participant age, clearer medical salience, and lower perceived burden of engagement. In contrast, ED‐focused interventions often require more intensive and emotionally salient self‐monitoring, which may contribute to greater perceived burden and higher dropout risk, particularly in longer or more demanding protocols. Importantly, differences in outcome measurement should also be considered when interpreting findings across conditions. Obesity‐related outcomes, such as body weight, are typically assessed using standardized, continuous, and objectively quantifiable measures, whereas ED‐related outcomes (e.g., binge‐eating or compensatory behaviors) are often assessed via self‐report or expert interview and may be more episodic, context‐dependent, and subject to greater reporting variability. Overall, these findings support the feasibility and acceptability of EMI‐based digital interventions across ED and obesity population, consistent with prior research demonstrating the utility of digital self‐monitoring and feedback tools in behavioral treatment settings (Burke et al. 2011; de Zwaan et al. 2017; Hilbert et al. 2019). These findings also support the rationale for examining EDs and obesity within a unified framework. Although the two conditions differ in nature, the partially overlapping self‐regulatory and behavioral processes targeted by EMIs—such as responses to emotions, cravings and environmental cues (e.g., Leppanen et al. 2022; Presseller et al. 2022)—may help explain why, despite differences in clinical presentation and treatment goals, similar intervention principles can be applied to these populations.

Recent reviews have highlighted the growing use of momentary and adaptive technologies in obesity and ED‐related interventions (e.g., Koh et al. 2025; Rapelli et al. 2025). At the same time, although EMA research has substantially advanced the identification of momentary risk factors for maladaptive eating behaviors, the translation of these insights into intervention design remains limited (Heron and Smyth 2010; Nahum‐Shani et al. 2017). Across the studies included in the present review, many EMI approaches primarily relied on enhanced self‐monitoring, reminders, and feedback, whereas relatively few implemented fully adaptive or context‐sensitive intervention architectures informed by real‐time EMA data. Thus, despite increasing interest in JITAI frameworks, highly responsive momentary interventions remain relatively uncommon in routine clinical care. Future research should focus not only on increasing technological sophistication, but also on identifying clinically feasible ways to integrate EMA‐derived insights into scalable and personalized intervention delivery.

### 4.3 Clinical Outcome and Interventions

#### 4.3.1 EDs

Across studies targeting EDs, EMI‐augmented interventions generally produced significant within‐group improvements in binge‐eating frequency and ED psychopathology from baseline to post‐treatment. Among the six included ED trials, five implemented EMIs within CBT‐based treatment contexts (Hilbert et al. 2025; Juarascio et al. 2021, 2022; Juarascio, Presseller, Srivastava, et al. 2023; Shingleton et al. 2016), while one evaluated a stand‐alone text messaging intervention (Shapiro et al. 2010). Across adjunctive trials, symptom improvements were primarily reflected in within‐group effects, with inconsistent evidence for superiority over active CBT‐based control conditions, particularly at post‐treatment.

Beyond acute symptom change, accumulating evidence suggests that feedback‐driven and context‐sensitive EMI components may contribute more to treatment engagement, skill consolidation, and maintenance of behavior change, rather than for immediate symptom reduction alone. This pattern is most clearly illustrated in adaptive or just‐in‐time EMI trials, where benefits emerged during periods of reduced therapeutic contact or follow‐up rather than during the structured treatment (e.g., Juarascio, Presseller, Srivastava, et al. 2023), and in withdrawal‐design evidence demonstrating within‐participant improvements in restriction‐related processes without sustained between‐condition symptom superiority (Juarascio et al. 2022). Although EMI‐augmented CBT can yield substantial post‐treatment symptom reductions, superiority over CBT alone is often attenuated after baseline or at follow‐up (Hilbert et al. 2025), indicating context‐dependent rather than durable comparative effects. Clinically, automated, feedback‐based EMI components embedded within CBT may help bridge the transition from structured therapy to more autonomous self‐regulation, reinforcing therapeutic strategies in everyday contexts and potentially reducing vulnerability to early relapse. This interpretation is consistent with prior conceptual and empirical work suggesting that EMIs may function as maintenance‐oriented extensions of evidence‐based ED treatments, by promoting continued engagement and skill application beyond the clinical setting (Levinson et al. 2018). Overall, sustained post‐treatment superiority over CBT remains insufficiently supported, partly due to limited long‐term follow‐up assessments, and heterogeneous abstinence‐based outcome definitions.

By contrast, Shingleton et al. (2016) found that personalized motivational text messages delivered alongside group CBT did not sustain post‐treatment reductions in binge‐eating or dietary restraint, suggesting that motivational prompts alone—even when individualized—may be insufficient for durable behavioral change in adults with binge‐eating‐related disorders. This observation appears to contrast with motivational interviewing–based frameworks, which emphasize the limited effectiveness of directive guidance for facilitating behavior change (Miller and Rollnick 2013). One possible explanation is that individuals receiving EMI‐augmented CBT may have already undergone motivational clarification during therapy, thereby reducing the incremental value of additional motivational prompts in daily life (Fairburn 2008; Linardon et al. 2020). In this context, directive behavioral suggestions delivered via digital devices may function less as interpersonal control and more as structured self‐regulation support, consistent with models of just‐in‐time adaptive and digitally supported behavior regulation (Nahum‐Shani et al. 2017). Evidence from other digital health domains similarly suggests that prescriptive informational formats providing explicit behavioral guidance can enhance patient engagement (McClure et al. 2013). Taken together, these findings suggest that the apparent divergence from motivational interviewing–based frameworks may reflect prior motivational clarification during therapy, and that directive, behaviorally specific EMI components delivered via digital systems may be particularly beneficial when intrinsic motivation alone is insufficient, particularly among individuals with chronic symptoms or impaired self‐regulation (McClure et al. 2013; Nahum‐Shani et al. 2017).

#### 4.3.2 Obesity

Among studies targeting obesity, EMI‐based interventions were associated with small but clinically significant weight reduction over the short to medium term (typically 3 to 6 months), with average within‐treatment reductions generally in the range of 1–3 kg or 1–2 kg/m^2^ BMI. Although between‐group differences were often small, EMI conditions more consistently facilitated adherence to behavioral targets, including dietary self‐monitoring, PA, and goal tracking compared with less interactive digital formats. The weight loss observed in EMI studies was smaller than that typically achieved by traditional face‐to‐face behavioral weight loss programs, which generally produce reductions of approximately 5%–10% of initial body weight within the first 6–12 months (Spring et al. 2017; Thomas et al. 2019). However, EMI‐based interventions tended to yield greater weight loss than standard eHealth programs without ecological or adaptive components, which often produce only 1–2 kg or 1%–3% weight reduction over similar periods (Patel et al. 2019). This pattern aligns with prior systematic reviews indicating that fully digital or remote eHealth programs typically yield smaller weight loss effects than face‐to‐face behavioral interventions (Block et al. 2015; Turner‐McGrievy and Tate 2011). These findings suggest that while EMI cannot fully replicate the effects of intensive face‐to‐face programs, including in more adaptive just‐in‐time intervention designs, it outperforms most static digital interventions by providing more frequent, contextually relevant and adaptive feedback. This enhanced responsiveness may improve self‐regulation and adherence, highlighting EMI's broad potential as a middle path that balances scalability with clinical significance.

However, weight‐loss effects observed in EMI‐based interventions tended to attenuate by 12 months, a pattern that is consistent with the broader weight‐loss literature, where initial treatment gains commonly diminish over time (LeBlanc et al. 2018). Multiple trials indicate that although personalized and adaptive feedback, or coach‐assisted EMI may yield more pronounced weight loss in the short term than self‐monitoring or control groups, these between‐group differences were not maintained at later assessment points (e.g., around 12 months, Burke et al. 2022; Svetkey et al. 2015; van Beurden et al. 2019). This pattern may suggest that self‐monitoring itself represents a potent active ingredient for behavior change and that, in terms of weight loss, EMIs may not consistently confer substantial benefits beyond structured self‐monitoring alone, with emerging evidence indicating that effects may be context‐dependent rather than uniformly superior. Accordingly, emerging evidence suggests that the added value of EMIs may depend less on the delivery modality and more on the quality, timing, and personalization of feedback, consistent with design principles emphasized in prior EMI research (Klasnja et al. 2018; Nahum‐Shani et al. 2017). When both conditions incorporate structured behavioral strategies, as observed in several obesity‐focused trials (e.g., Svetkey et al. 2015; Burke et al. 2022; van Beurden et al. 2019), improvements in both groups over time may reduce the incremental effects of added feedback, underscoring both the durability of self‐monitoring and the broader challenge of sustaining weight loss once external support is withdrawn. van Beurden et al. (2019) demonstrated that beyond weight loss, both loss of control and binge‐eating episodes decreased, suggesting that EMIs may influence eating regulation mechanisms even with minimal weight change. In the study by Mundi et al. (2015), participants for weight‐loss surgery achieved an average preoperative weight reduction of 5.6% of baseline weight during the EMI‐supported intervention period and reported higher levels of vigorous (i.e., higher‐intensity) PA, which was interpreted as a positive outcome in the context of obesity‐focused treatment. Notably, early responsiveness to EMI correlated with higher exercise confidence and more vigorous activity levels over the preoperative period. This finding supports potential pathways through which EMI may improve behavioral outcomes by enhancing engagement, fostering self‐efficacy, and promoting sustained self‐regulation.

Although retention tends to be higher in obesity‐focused trials, this disparity should not be interpreted as evidence of superior therapeutic efficacy. Differences in clinical impact between EDs and obesity studies should also be interpreted in relation to the proximity of outcome measures. Outcomes frequently assessed in ED trials, such as binge‐eating frequency and ED psychopathology, are relatively proximate to the self‐regulatory processes targeted by EMI and may therefore show more immediate behavioral change. In contrast, obesity‐related outcomes, such as body weight and BMI, represent more distal endpoints that are less sensitive to short‐term behavioral changes and often require sustained intervention exposure and long‐term maintenance to yield enduring between‐group differences (LeBlanc et al. 2018). These findings also support the rationale for examining EDs and obesity within a shared intervention framework. Although the two conditions differ in clinical presentation and treatment outcomes, partially overlapping self‐regulatory and behavioral processes, such as responses to emotions, cravings, and environmental cues, may help explain why similar EMI principles can be applied across these populations. In ED studies, EMIs were most commonly implemented as adjuncts to CBT‐based psychotherapy, primarily reinforcing skill application between treatment sessions rather than functioning as standalone interventions. Across studies, EMI‐augmented interventions were generally associated with moderate to large within‐group reductions in binge‐eating and ED psychopathology, although evidence for superiority over active control conditions remained limited or inconsistent. In contrast, obesity‐focused EMIs were more often positioned as central components of digitally delivered behavioral weight‐management interventions, with greater emphasis on motivation, adherence, and behavioral self‐regulation than on rapid anthropometric change. Consistent with broader digital health literature, obesity‐focused interventions generally produced modest short‐ to medium‐term weight changes, suggesting that durable anthropometric improvement may require more intensive and sustained behavioral support (Balaskas et al. 2021). Future obesity‐focused EMI interventions may therefore benefit from incorporating structured maintenance components, such as adaptive feedback, relapse‐prevention strategies, and ongoing human or peer support, to facilitate sustained behavioral and physiological change.

### 4.4 Methodological Quality and Risk of Bias

Regarding methodological quality, the overall risk of bias was rated as low to moderate in RCTs and moderate to high in non‐randomized studies. The most frequent sources of bias were confounding and missing data, whereas bias related to participant selection, outcome measurement, and selective reporting was generally low. These methodological shortcomings, combined with the short follow‐up durations of some trials, limit the reliability of conclusions and highlight the need for more rigorous, adequately powered studies with longer follow‐up periods, transparent reporting, and standardized outcome measures.

### 4.5 Strengths and Limitations

This systematic review uniquely provides a focused synthesis of treatment‐oriented EMIs applied in clinical populations with EDs and obesity. A comprehensive search across eight databases and rigorous eligibility criteria ensured inclusion of interventions that met established EMI definitions, clearly distinguishing EMIs from related approaches such as EMA and general eHealth tools. To enhance methodological rigor, all full‐text screening records were independently rated by two reviewers, with disagreements resolved through discussion within the research team. Risk of bias was assessed for all included studies. The review was preregistered.

However, some limitations need to be considered when interpreting the findings. First, the number of EMI trials targeting EDs remained relatively small, and diagnostic representation was limited despite the inclusion of several randomized studies. Methodological rigor also varied across studies, with some studies employing non‐randomized or uncontrolled designs, thereby limiting causal inferences and increasing susceptibility to selection and confounding bias. Furthermore, conclusions regarding long‐term efficacy of EMI remain preliminary due to limited follow‐up periods and inconsistent reporting of post‐treatment outcomes.

Participant diversity was also limited, as most samples comprised predominantly White, female, middle‐aged adults, restricting generalizability. This limitation may also influence the applicability of findings, as engagement with EMI components and responsiveness to real‐time feedback may vary across demographic and cultural contexts. For example, individuals from underrepresented racial or ethnic groups may face barriers related to access to digital technologies, digital literacy, or the cultural relevance of intervention content, which may reduce engagement or intervention effectiveness (Latulippe et al. 2017; Veinot et al. 2018). Prior research similarly suggests that engagement with digital interventions may vary across populations, indicating that current findings may overestimate engagement and feasibility in more diverse populations (Perski et al. 2017). Gender differences in symptom reporting and help‐seeking behaviors may further influence how individuals interact with and benefit from EMI‐based interventions. Furthermore, retrospective assessment of eating‐related outcomes may introduce recall bias, particularly with longer recall windows, whereas real‐time EMI assessments offer greater ecological validity and more temporally sensitive data on eating behaviors.

Another limitation of the included ED research is that all interventions implemented EMI as adjuncts to CBT‐based psychotherapy. Consequently, the efficacy of standalone EMI interventions, as well as the independent contributions of specific EMI components within multicomponent treatments, remain unclear. Future research should evaluate standalone EMI interventions and consider component‐level or factorial designs to clarify underlying mechanisms of change (Juarascio, Presseller, Srivastava, et al. 2023; Juarascio, Presseller, Trainor, et al. 2023).

Finally, reliance on BMI as the primary criterion for defining obesity across the included studies represents an important limitation of the existing EMI literature. BMI was consistently used to define eligibility and evaluate weight‐related outcomes; however, it does not directly capture body composition, fat distribution, or functional health impairment, and may not fully reflect individual differences in adiposity‐related health risk across demographic groups (Rubino et al. 2025). Given that many included samples were predominantly female, reliance on BMI alone may oversimplify variability in obesity‐related clinical presentations and health risk.

Regarding the strengths and limitations of the present review itself, stringent inclusion criteria ensured that only clearly defined EMIs were analyzed, although this also reduced the number of eligible studies and excluded emerging digital interventions incorporating partial EMI components. No language restrictions were applied during the search; however, all eligible studies were published in English, which may still limit the global representativeness of the findings. In addition, several EMI studies remain at the protocol or early feasibility stage, indicating that the evidence base is still emerging. Substantial heterogeneity in interventions, outcomes, and follow‐up durations similarly impeded quantitative synthesis. As the evidence base matures and standardization progresses, future reviews may enable meta‐analytic assessments of intervention effectiveness.

### 4.6 Implications and Future Directions

Despite these limitations, existing evidence suggests that EMIs primarily function as adjunctive supports to established treatments for EDs and obesity, enabling the real‐time application of therapeutic skills within everyday contexts. In EDs, EMIs were most commonly integrated into CBT‐based psychotherapy, whereas obesity‐focused interventions were typically incorporated into BWL programs, consistent with emerging smartphone‐assisted care models (Hilbert et al. 2023). Although the independent therapeutic contribution of EMIs beyond existing treatment frameworks remains unclear, their scalability and low‐intensity delivery suggest potential utility within stepped‐care, maintenance‐phase, and relapse‐prevention approaches. Nevertheless, the proposed benefits of EMIs for self‐regulation, treatment adherence, and relapse prevention remain insufficiently examined, as relatively few studies directly evaluated these mechanisms as moderators or mediators of treatment outcomes (Presseller et al. 2022).

Future research should prioritize adequately powered RCTs, standardized outcome measures, and extended follow‐up periods to clarify durability, generalizability, and real‐world effectiveness of EMI interventions, and to determine whether specific EMI components—such as real‐time feedback, contextual prompting, or skill reinforcement—meaningfully translate into behavioral and clinical change (Juarascio, Presseller, Srivastava, et al. 2023; Juarascio, Presseller, Trainor, et al. 2023). In addition, trials evaluating EMIs as low‐threshold or stand‐alone interventions may help define their optimal clinical positioning within evolving digital mental‐health care pathways.

## Author Contributions

**Emily K. Presseller:** methodology, formal analysis, writing – review and editing, validation. **Anja Hilbert:** conceptualization, methodology, supervision, writing – review and editing. **Wenlu Mao:** conceptualization, methodology, investigation, data curation, formal analysis, writing – original draft, writing – review and editing. **Ricarda Schmidt:** methodology, investigation, writing – review and editing. **Adrienne Juarascio:** conceptualization, methodology, writing – review and editing.

## Funding

The authors have nothing to report.

## Conflicts of Interest

Emily K. Presseller reports funding from the National Institutes of Health (Grant No. DP5OD039510). Dr. Anja Hilbert reports receiving research grants from the German Federal Ministry of Education and Research, German Research Foundation, Innovation Fund, and Roland Ernst Foundation for Health Care; royalties for books on the treatment of eating disorders and obesity with Hogrefe and Kohlhammer; honoraria for workshops and lectures on eating disorders and obesity and their treatment, including for Lilly, Novo Nordisk, and Rhythm; honoraria as editor of the International Journal of Eating Disorders; and honoraria as a consultant for Takeda and Medice. No other competing interests were reported. Dr. Anja Hilbert is an editor of the International Journal of Eating Disorders and co‐author of this article. She was excluded from editorial decision‐making related to the acceptance of this article for publication in the journal. The other authors declare no conflicts of interest.

## Supporting information


**Table S1:** Overview of extracted data from included studies.
**Table S2:** Operational definitions of key terms and abbreviations used in this review.

## Data Availability

Data sharing not applicable to this article as no datasets were generated or analysed during the current study.
