# Supplementary material for: Ecological Momentary Interventions in the Management of Eating Disorders and Obesity: A Systematic Review
Source: Int J Eat Disord. 2026 Jun 2;59(10):2119–47. doi: 10.1002/eat.70140 (PMC13655985; doi:10.1002/eat.70140)
Supplement: Supplementary file 1 [file EAT-59-2119-s001.docx]

**Ecological Momentary Interventions in the Management of Eating Disorders and Obesity: A Systematic Review**

Wenlu Mao, Ricarda Schmidt, Emily K. Presseller, Adrienne Juarascio, Anja Hilbert

**Online supplement**

| Table S1 | Overview of Extracted Data from Included Studies |
| --- | --- |
| Table S2 | Operational Definitions of Key Terms and Abbreviations Used in This Review |

**Table S1** *Overview of Extracted Data from Included Studies*

| Category | Data to be extracted | Definition/ Description |
| --- | --- | --- |
| Study Characteristics | First author; year of publication; country; study design (e.g., RCT, non-RCT, uncontrolled intervention study); duration of interventions; follow-up length | Basic bibliographic and methodological information of each study |
| Participant Characteristics | Sample size (*n*), mean age (*SD*); gender distribution (% female); nationality; education level; BMI (mean, *SD*); diagnostic status (EDs or obesity) | Demographic and clinical baseline characteristics of participants |
| EDs and Obesity Assessment | Diagnostic instruments; screening tools; outcome measures; assessment timing (baseline, post, follow-up) | Clinical or validated tools used for diagnosis and outcome evaluation |
| EMIs Methodology | Type and format of EMI (e.g., smartphone app, SMS, wearable); components (feedback, prompts, self-monitoring, JITAIs); frequency and duration; delivery mode; tailoring or contextualization | Core technical and behavioral design features of the EMI intervention |
| Outcome | ED outcomes (e.g., binge episodes, compensatory behaviors, EDE scores); obesity outcomes (e.g., weight loss, % fat, WHR, metabolic markers); acceptability; adherence; retention; adverse events | Primary and secondary outcomes, including clinical effectiveness and feasibility indicators |

*Note.* BMI = body mass index; ED = eating disorder; EDE = eating disorder examination; EMI = ecological momentary intervention; n = number; Non-RCT = non-randomized controlled study; RCT = randomized controlled study, SD = standard deviation; WHR = waist-to-hip ratio.

The extracted information was organized and managed using Excel for data compilation and synthesis.

**Table S2** *Operational Definitions of Key Terms and Abbreviations Used in This Review*

| Acceptability | Participants’ subjective evaluation of the intervention, including satisfaction, perceived usefulness, and willingness to continue use. |
| --- | --- |
| Adherence | The degree to which participants engaged with the EMI as prescribed by the study protocol. |
| Ecological momentary assessment (EMA) | Repeated, real-time data collection in naturalistic settings to capture momentary emotional, behavioral, or clinical states. |
| Ecological momentary intervention (EMI) | Interventions delivering treatment content or feedback in real time, tailored to individuals’ current states, behaviors, or contexts, primarily via mobile or digital devices. |
| Engagement | Behavioral interaction with the EMI, reflected by objective or self-reported usage indicators (e.g., response rates to prompts, frequency of app use), considered a component of adherence. |
| Just-in-time adaptive intervention (JITAI) | A subtype of EMI that uses real-time data and prespecified decision rules or algorithms to dynamically adapt intervention content, timing, or intensity. |
| Retention | The proportion of participants who completed post-treatment assessments and provided outcome data at the end of the intervention. |
